# Molecular epidemiology of pregnancy using omics data: advances, success stories, and challenges

**DOI:** 10.1186/s12967-024-04876-7

**Published:** 2024-01-26

**Authors:** Ali Rahnavard, Ranojoy Chatterjee, Hui Wen, Clark Gaylord, Sabina Mugusi, Kevin C. Klatt, Emily R. Smith

**Affiliations:** 1 Computational Biology Institute, Milken Institute School of Public Health, The George Washington University, Washington, DC 20052 USA; 2 Department of Biostatistics and Bioinformatics, Milken Institute School of Public Health, The George Washington University, Washington, DC 20052 USA; 3 Department of Clinical Pharmacology, Muhimbili University of Health and Allied Sciences, Dar es Salaam, Tanzania; 4 Nutritional Sciences & Toxicology, University of California, Berkeley, CA 94720 USA; 5 Department of Global Health, The Milken Institute School of Public Health, The George Washington University, Washington, DC 20052 USA; 6 Department of Exercise and Nutrition Sciences, The Milken Institute School of Public Health, The George Washington University, Washington, DC 20052 USA

**Keywords:** Pregnancy, Omics, Epidemiology, Computational biology

## Abstract

Multi-omics approaches have been successfully applied to investigate pregnancy and health outcomes at a molecular and genetic level in several studies. As omics technologies advance, research areas are open to study further. Here we discuss overall trends and examples of successfully using omics technologies and techniques (e.g., genomics, proteomics, metabolomics, and metagenomics) to investigate the molecular epidemiology of pregnancy. In addition, we outline omics applications and study characteristics of pregnancy for understanding fundamental biology, causal health, and physiological relationships, risk and prediction modeling, diagnostics, and correlations.

**Supplementary Information:**

The online version contains supplementary material available at 10.1186/s12967-024-04876-7.

## Background

Pregnancy represents a dynamic physiological state aimed at supporting the growth and development of the fetus, as well as the primary additional organ of pregnancy, the placenta. To date, researchers have characterized broad physiological changes associated with pregnancy throughout the cardiovascular [1–3], respiratory [4], renal [5], immunological, and endocrine systems. Numerous changes include alterations in metabolic rate, insulin sensitivity, body composition, increased blood volume, reduced circulating albumin, increased cardiac output, increased lung tidal volume and reduced functional capacity, and increased glomerular filtration rate.

While reasonably well-characterized at the physiological level, precise cellular and molecular mediators of such changes remain an active and challenging area of study. Characterizing underlying mechanisms is paramount, not only as it improves our understanding of basic biology but also in understanding factors that influence risk of pregnancy-specific diseases. Indeed, pregnancy is a unique physiological stressor with the potential to cause, exacerbate, or uncover disease. Common disorders unique to pregnancy include hyperemesis gravidarum, gestational diabetes mellitus, and hypertensive disorders of pregnancy, such as preeclampsia. Understanding the range of normal variation within healthy human pregnancies and molecular changes that underlie diseases of pregnancy are key to improving screening, diagnosis, prevention, and treatment.

**Fig. 1:**
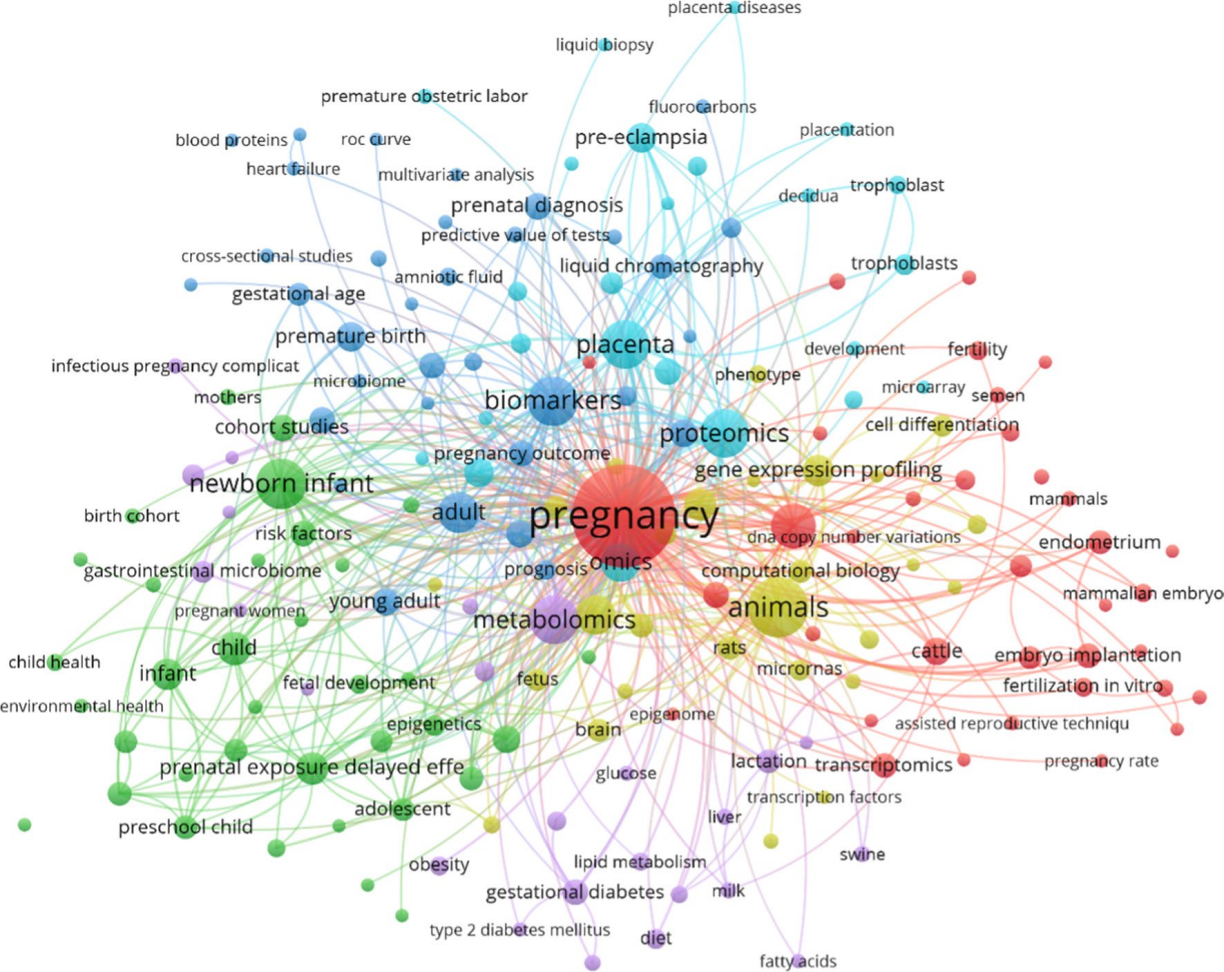
Current important scientific directions of omics utilization in pregnancy. We processed the abstracts of 428 papers identified in our search of omics and pregnancy and conditions we processed. From 2020 extracted scientific keywords from abstracts that occurred with pregnancy and omics, we show 181 keywords with at least 5 co-occurrences. The co-occurrence link with other keywords is also measured and shown as links between keywords. Colors represent general topics and technology used, for example, blue for animals and green for pregnancy, etc.". The network analysis was performed by VOSviewer [6]. Nodes are keywords that are linked by edges for their co-occurrence. Edges between two keywords in the graph reflect the number of co-occurrences

High throughput technologies, coupled with powerful bioinformatic pipelines, allow for the molecular profiling and quantification of biological systems at the levels of gene expression (transcriptomics), proteins (proteomics), metabolites (metabolomics), the microbiota (microbiome), and the epigenome (epigenomics) and greatly expand our capacity to identify causal factors and correlates of the physiological changes that accompany pregnancy. We undertook text mining literature (Fig. 1) to assess how omics approaches have been applied across pregnancy and infant health (e.g., preeclampsia and gestational diabetes), clustered (colors) by co-occurrence and progress overtime colored by year of publication (Additional file 1: Fig. S1).

**Fig. 2:**
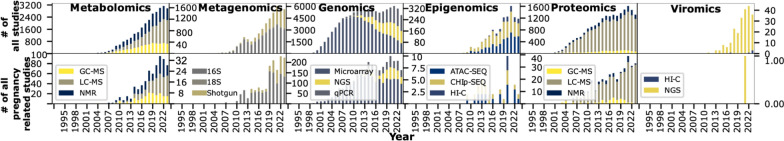
Omics advancements featured in **Row 1** literature overall, and **Row 2** pregnancy investigation. Omics technology usage continues to grow in research studies. The number of publications by year with omics keywords and pregnancy in their abstract extracted from the PubMed database is shown in panel **Row 2**. The colors in the stacked bar charts show the trends of different specific omic technologies. For example, gas chromatography-mass spectrometry (GC–MS) has always been used in metabolomics. However, the use of liquid chromatography-mass spectrometry (LC–MS) is more common in recent years; for proteomics, mass spectrometry continues to be the dominant technique. This figure was generated with pubSight [8]

Overall literature use of omics is shown (Additional file 1: Fig. S2) using PubMed Database [7] for searches, and the search strategy is described in Supplementary Methods. Searches for specific technologies used across different omics approaches have increasingly been used to investigate biology in general (Fig. 2—row 1) and a limited number of studies, including pregnancy (Fig. 2—row 2). Herein, we describe the promise and potential of utilizing omics technologies to study pregnancy, their existing contribution to our understanding of pregnancy-related physiology and adaptation, and both future research needs and challenges in this area of study.

## Main text

### Review method

We investigated and summarized various aspects and applications of omics technologies used in pregnancy research through narrative-focusing case studies detailing how omics have been used successfully to inform our understanding of both fundamental aspects of pregnancy physiology and disease. We further provide critical commentary on the need for investment in large cohorts designed for studying pregnancy physiology and the employment of diverse omics approaches employing recent advances in data science.

To inform qualitative synthesis of the existing literature base, we searched PubMed using search terms (Additional file 1: Table S1) to identify pregnancy-related research articles employing different types of omics, documented the biological hypotheses identified by the paper, year of publication, number of citations, and extracted the following elements in each study or a group of related studies and then incorporated them into the review: (1) study design, (2) omics type and corresponding profiling technologies, (3) main hypothesis or discovery, (4) application, (5) computational and analytical approaches used and (6) challenges and suggestions for future work. We used publication year and type of omics used in the study for quantitative synthesis of information. We compared the reported findings for quality control of discoveries (Additional file 2).

### Omics success stories in pregnancy research

#### Genomics: single-cell sequencing of the placenta

Omics profiling of the placenta has greatly advanced our understanding of the cellular composition of the placenta. Gross placental anatomy typically classifies the organ as containing decidual tissue, a junctional zone, the labyrinth, the chorionic plate, and amniotic plate. These macroscopic structures are composed of several placental cell types, including cytotrophoblasts, syncytiotrophoblasts, extravillous trophoblasts, various stem cell populations, stromal cells, and immune cells. The diversity of cell types of both maternal and fetal origin, and their interface, represent a complex dynamic, necessitating high throughput technologies to characterize them simultaneously. Several groups have recently employed single-cell sequencing techniques, relying on microfluidic and droplet-based technologies and advanced Ribonucleic Acid (RNA) sequencing, to describe unique cell type signatures at the maternal–fetal interface in both health and disease [9–11]. These signatures include non-proliferative cytotrophoblasts in the villi and basal plate (not in chorioamniotic membranes) [11] and new lymphatic endothelial cells in the decidua, primarily in the decidua parietalis of chorioamniotic membranes providing a major route for maternal lymphocytes (e.g. T cells) infiltrating the maternal–fetal interface. Large differences in gene expression profiles of cell types found in more than one location in the placenta demonstrate microenvironment sub-niches of cell types [11].

#### Placental multi-omics for understanding preeclampsia

The placenta is a great source of biomarkers because it is associated with maternal blood during pregnancy and provides the fetus with immune protection and nutrient delivery [12]. Profiling multi-omics on samples from a placenta can lead to understanding pregnancy complications such as preeclampsia. Preeclampsia (PE) is a life-threatening hypertensive condition affecting 3–5% of all pregnancies and often leads to serious, sometimes fatal, complications for both mother and baby. Specifically, ongoing multi-omic research has shed light on predicting or screening for the condition, understanding the mechanism of the disease, and identifying treatments.

One study used such techniques to discover the possible markers or factors that can lead to onset of preeclampsia. As such, the researchers combined several studies and generated a dataset of samples from 173 patients, 77 of whom had PE [13]. 3,663 genes were identified from the combined datasets using genomics and metabolomics. After filtering for invariant genes, a total of 14,653 were used to identify two clusters for controls and one cluster for PE samples. This study marks a monumental step forward in PE research and can be used as a basis to conduct further research studies regarding gene expression and analysis of PE markers, which can lead to creating proper screening protocols and testing to ensure the safety and well-being of patients susceptible to PE.

Omics profiling has played a key role in identifying novel, causal pathophysiological factors in preeclampsia. Phenotypes of PE resolve following parturition, and it is generally accepted that circulating factors released by the placenta cause PE. In PE, trophoblasts fail to adequately invade the maternal decidua and remodel maternal spiral arteries, leading to placental ischemia and, ultimately, maternal and fetal disease. The use of early high throughput RNA transcriptome profiling techniques, known as microarrays, proved useful in identifying secreted factors overproduced by the placenta in PE that contribute to the high-risk maternal disease phenotype. Placentas were taken at term and led to future experimental evidence identifying the role of soluble fms-like tyrosine kinase 1 (sFLT1) and soluble endoglin (sENG) in the pathogenesis of PE [14].

Another study used omics to investigate the pathophysiology of preeclampsia, which was largely unknown at the time [14]. The results of this technique on the placental tissue of preeclampsia women and women with no preeclampsia showed the upregulation of the soluble fms-like tyrosine kinase 1 (sFLt1) gene. This knowledge allowed them to carry out further experiments, including genomics techniques such as Northern blot analysis and Western blot analysis, both of which are used by researchers to detect and quantify RNA and proteins, respectively. Their study found that the upregulation of sFLt1 downregulated vascular endothelial growth factor (VEGF) and placental growth factor (PLGF). VEGF and PLGF promote angiogenesis, and their downregulation is associated with endothelial dysfunction, promoting preeclampsia. Aspirin is sometimes prescribed as a treatment for preeclampsia, but it has been shown to not have any significant effect on sFLt1 levels [15]. It is shown that aspirin is associated with a decrease in sFLT1 expression [16, 17]. Gene expression knowledge continues to guide scientists in search for what can control the levels of sFLt1 for effective treatment of preeclampsia. For example, ABCC8, KCNJ11, and ADAM12 are other genes implicated during pregnancy in a case of placental abruption [17, 18].

### Metabolomics biomarkers for predicting abruption risk

Metabolism is an essential process during pregnancy, and understanding the metabolic events that occur during pregnancy is critical to investigating fetus and mother health. A study involving 30 Danish women performed untargeted metabolomic (LC–MS) profiling on 784 blood samples [19] and investigated the dynamic of metabolites during pregnancy and reported metabolites biomarkers as indicators for labor occurrence and five metabolites that time gestational age is confirmed with ultrasound results. Metabolites in placental abruption (PA) were investigated in a case–control study with 51 case and 51 control maternal serum samples [20] using LC–MS spectroscopy. The study showed that along with symptoms such as early pregnancy vaginal bleeding, metabolites including dodecanoylcarnitine/ dodecenoylcarnitine (C12 / C12:1) and phosphatidylcholine acyl-alkyl C 38:1 (PC ae C38:1) are biomarkers for predicting abruption risk.

### Proteomics to investigate biomarkers of preeclampsia

Proteomics has become one of the most important applications in biomedical research. Multiple studies have used proteomics to find biomarkers that can predict preeclampsia [21–24], fetal growth restriction (FGR) [23, 25], gestational diabetes mellitus [26, 27], and preterm delivery [28, 29].

Proteomics techniques were used in a longitudinal study with a nested case–control setup [21] and found matrix metalloproteinase-7 and glycoprotein IIbIIIa complex as the most reliable predictors of preeclampsia at 1–22 weeks of gestation. Elevated sialic acid binding immunoglobulin-like lectin 6 (siglec-6) and activin-A were the best predictors of the subsequent development of early preeclampsia at 1–28 gestational weeks [25]. The activated leukocyte cell adhesion molecule, siglec-6, and VEGF-121 at 1–32 weeks [30] and increased siglec-6, activin-A, and VEGF-121 at 1–28 weeks can also be one of the predictors of preeclampsia. Another study also indicated that α-1-antichymotrypsin (SERPINA3) has increased 2 to 3 times in plasma prior to preeclampsia [30]. PE can stimulate inflammation and damage the pro-inflammatory cytokines and substances, whereas SERPINA3 inhibits protease growth [31, 32]. That is, the inflammatory response of SerpinA3 may be related to the pathophysiology and pathogenesis of PE [32]. While most studies use plasma or placental tissue as sample matrix, interestingly, one used cerebrospinal fluid to discover the predictive biomarkers to discriminate against women with severe preeclampsia [22]. They have found that those women with severe preeclampsia have nanomolar amounts of free hemoglobin in their CSF compared to those who have mild preeclampsia or normal pregnancy.

Proteins also play an essential role as biomarkers of pregnancy. There are four human pregnancy-associated plasma proteins in the third trimester: PAPP-A, PAPP-B, PAPP-C, and PAPP-D. Pregnancy-associated plasma protein A (PAPP-A), also known as Pappalysin-1, is a high-molecular-weight glycoprotein produced by the placenta [33]. PAPP-A concentration measurement can help detect some pregnancy complications such as Down’s syndrome, preeclampsia, and gestational diabetes during pregnancy [34]. A past study reported that the median value of PAPP-A in the first trimester in the abnormal fetus chromosome pregnancy group is significantly lower than normal pregnancy group [35]. Pregnant women who have lower PAPP-A concentrations have higher risk of preeclampsia [36]. A case–control study showed a significantly lower PAPP-A concentration in the gestational diabetes group compared to the normal pregnancy group in the first trimester. They also pointed out a significant difference in PAPP-A median between those who have pregnancy complications and those who do not have complication groups in the gestational diabetes group, whereas there is no such difference in PAPP-A median between complications and non-complication in the normal pregnancy group [37]. These studies indicated the clinical significance of detecting PAPP-A concentration.

A serum peptide analysis-liquid chromatography mass spectrometry was conducted to identify serological markers capable of diagnosing preeclampsia. Among the 19-peptide biomarker panels, all of them indicated the potential efficacy of predicting preeclampsia with high sensitivity and specificity [38].

### Multi-omic of pregnancy microbiome

Several multi-omic studies may leverage the Integrative Human Microbiome Project [39], a data repository of many data collection efforts focused on understanding the human microbiome via various omics techniques. Related to this effort is the Multi-omic Microbiome Study—Pregnancy Initiative [40] (MOMS-PI), with 90 women (49 of African ancestry, 41 of European ancestry) forming the MOMS-PI Term Birth cohort. One effort, MOMS-PI demonstrates differences between the microbiome and pathways of African and non-African-descent pregnant women [41]. Among the results of this effort, African-descent women have a higher prevalence of bacterial vaginosis-associated bacteria (BVAB) [42], a pathogen related to preterm delivery and reduced birth weight. During pregnancy, the microbiome of these women undergo greater change than those of the non-African-descent cohort, and the two cohorts become more similar. Based on profiles generated from metagenomic and metatranscriptomic data, during pregnancy, the incidence of BVAB goes down in preference to microbiota associated with more successful pregnancies, notably Lactobacillus. While focused on metagenomic and metatranscriptomic analyses, this effort clearly demonstrates the utility of omics methods in understanding pregnancy health.

### Multi-omics to study Fetal Growth Restriction (FGR)

Proteomics can also be used to investigate fetal growth [23]. In a case–control (10 FGR/10 normal) study [23] using high-precision LC–MS (Thermo-fisher Q-Exactive Orbitrap), 95 differentially expressed proteins of human placenta were compared between the two groups. Among these 95 proteins, 35 are related to two major molecular networks: erythropoiesis (hemoglobin network) and oxidative stress (nicotinamide adenine dinucleotide phosphate (NADPH) oxidase network), which play an important role in the pathological changes observed in FGR placentas. Specifically, NADPH oxidase, low-density lipoprotein (LDL), and SERPINA1 are associated with oxidative stress. HBA1, HBA2, HBG1, HBG2, and HBB, which are associated with erythropoiesis, are observed to increase and, therefore, could be among the causes of FGR.

Another case–control study with fewer participants (5 FGR/5 normal) used one of the proteomics techniques, tandem mass spectrometry (2D nano LC–MS/MS) analysis, to demonstrate the causal health and physiological relationships [25]. Through proteomics analysis, 25 out of 688 proteins were significantly differentially expressed between the uncomplicated pregnancy group and the fetal growth restriction group, with 16 decreased in abundance and 9 increased among the FGR group. The researchers concluded that compared to healthy pregnancies, lipid metabolism of those who are complicated by late-onset FGR may be disturbed. Protein–protein interaction network indicated that NOTCH1 could be an important regulator of the observed profile. The gene ontology analysis revealed that the efflux of cholesterol and phospholipids is mostly related to the top canonical pathways and biological processes in late-onset FGR.

### Microbiome role in glucoregulation and gestational diabetes mellitus

Advanced microbial species profiling has enabled understanding of the gut microbiome's influence on well-characterized physiological adaptations during pregnancy, such as relationship between pregnancy and gestational diabetes mellitus (GDM). The resistance to insulin is directly related to fetus development. This deregulation has been predominantly associated with the disruption of gut microbiome, which is directly known as denouement of pregnancy. Using the combination of discovery and targeted proteomics, it has been revealed that afamin and SAMP could be predictors of gestational diabetes mellitus [26]. Vitronectin is also demonstrated as the novel independent predictor of GMD. iTRAQ quantitative proteomics determined four proteins with high sensitivity and specificity that could help with early screening of GDM: APOE, F9, FGA, and IGFBP5 [27]. In addition, maternal tissues exhibit greater resistance to insulin signaling in the latter half of pregnancy [43]. Such a decrease in insulin sensitivity does not necessarily result in overt disease (e.g., gestational diabetes mellitus) but is likely an adaptive mechanism of pregnancy to ensure a significant fuel supply (i.e., fatty acids, glucose) available to the fetal compartment as fetal nutrient demands increase. Longitudinal profiling of the gut microbiome across pregnancy has demonstrated that there is a significant shift in its composition as pregnancy progresses, thus reducing the diversity which can be seen in a non-pregnant woman. Pregnancy is associated with a decrease in levels of Faecalibacterium [44] and increases in Bacteroides levels [45] linked with insulin shortage and obesity. The transfer of the microbes during the third trimester to mice induced overweight and a reduced level of insulin [46].

The microbiome diversity changes during pregnancy and these changes reflect health status, such as insulin reduction [46]. Insulin plays an important role in the development of the fetus. During the early gestational period, the production of insulin increases, but during the third trimester, insulin significantly decreases [46]. This phenomenon is due to fat storage during the early trimester and increases in endogenous glucose production during the third trimester [47, 48]. In general, microbiome diversity of mothers during pregnancy has been directly linked with the growth of the fetus [49, 50].

### Viromics: COVID-19 in pregnancy

Omics study reveals abnormal alterations of breastmilk proteins and metabolites in puerperant women (women who have just given birth) with COVID-19 [51]. Zhao and colleagues attempted to investigate whether breastmilk production is affected by COVID-19 [51]. Previous literature has well-established nutritional and non-nutritive components of breastmilk as crucial for the development of neonatal immune response among infants [52]. As a result, healthy nutritional components in breast milk from mothers are essential to sustaining healthy immunity and metabolism for infants via breastfeeding. Zhao et al. compare colostrum samples collected 3 days after delivery from four COVID-19 puerperant women and two healthy puerperant women operated with cesarean section. Colostrum samples were assessed by applying proteomics, lipidomics, and metabolomics analyses to profile the component alterations in breast milk of COVID-19 patients. In summary, proteomics and metabolomics uncovered significant alterations of numerous breast milk proteins and metabolites associated with COVID-19 puerperant women. The alterations of breastmilk components were suspected to be a reflection of the mother's physiological responses to COVID-19, which could have impacted their breast milk production and/or secretions from mammary glands. COVID-19 is suspected to affect the bacteria in the body of puerperant women, consequently altering bacterial metabolites that can be secreted into breast milk. Overall, the study suggests that maternal infection can influence breast milk composition and may have implications for infants.

### Pregnancy supplements and health outcomes

Various nutritional supplements have been associated with improving pregnancy outcomes in women, as well as long-term health outcomes in both women and infants. Omics tools have been involved in several of these discoveries and continue to improve pregnancy studies. Here we discuss some of these studies and supplements that have been implicated in them.

After previously demonstrating that fish oil supplementation during pregnancy decreases the risk of asthma and persistent wheezing in infants [53], untargeted liquid chromatography-mass spectrometry was used to profile the metabolomic sequence in the plasma samples of 6-month-old infants [54]. The analysis showed that the n-3 long-chain polyunsaturated fatty acids (n-3 LCPUFAs) found in fish oil affected the metabolome of the infant, and lower levels of metabolites related to the n-6 LCPUFA pathway and the tryptophan pathway were observed in these infants' metabolomes. There were also lower levels of saturated and monounsaturated long-chain fatty acid compounds but higher levels of tyrosine and glutamic acid pathway-related metabolites. This metabolic profile at age 6 months showed a strong association with the reduced risk of asthma observed in their previous study by age 5. The hypothesis that LCPUFAs implicate metabolic pathways was tested by using GC–MS as a standard research technique that aids in identifying and quantifying the metabolite content in a sample [55]. The polyunsaturated fatty acid (PUFA) concentration was quantified in the maternal plasma phosphatidylcholine (PC) fatty acid composition. Spirometry was used to measure lung function in the experimental and control groups. Their study found associations that suggest that maternal exposure to n-6 and n-3 fatty acids, found in fish oil, during pregnancy decreased the risk of wheezing or asthma in the infant. These studies applying omics tools explain the role of fish oil supplementation during pregnancy in improving maternal and infant health outcomes.

Another study of prenatal fish oil supplements used ultra-performance liquid chromatography-tandem mass spectroscopy (UPLC-MS/MS) [56], which is another omics tool applied for its high sensitivity and specificity. There was a decreased 14% risk of infants of mothers who took fish oil prenatal supplements developing asthma or persistent wheezing by age 3, versus the 28% risk observed in mothers who did not take this supplementation. Additionally, an increased level of active vitamin D in the maternal serum was associated with a significantly decreased risk of asthma and persistent wheezing in the health outcome of infants. The researchers concluded that LCPUFAs, found in fish oil, and Vitamin D_3_ are beneficial supplements for women during pregnancy to decrease the risk of asthma and persistent wheezing in their infants.

Common computational and statistical approaches used in pregnancy studies

Omics data are very powerful and can provide a detailed, molecular-level understanding of human health and, at the same time, very sensitive to bias due to measuring approaches and technologies. Utilizing omics requires technical replicates and repeated measurements over time for quality control and differentiating between noise and biological signals [39]. We summarize below studies using omics in the pregnancy context.

Case–control studies have been used to identify (1) metabolites, proteins, and microbes associated with the development of pregnancy-specific disease states, (2) consequences of complicated pregnancies on placental biology, (3) metabolites and proteins associated with developmental time points, reflective of maternal, placental or fetal metabolism, and (4) associations/exposure biomarkers linking metabolome/proteome/microbiome and reported environmental exposures (e.g., diet, pollutants, toxins, etc.). Various statistical and machine-learning methods have been used to quantify the dynamics of pregnancy omics. These analyses have mainly been used to identify biomarkers for specific health conditions or predict health status from omics profiles. In addition, pathway enrichment analysis has been performed to understand functional changes related to omics shifts. Differential abundance analyses are common tasks in most studies.

The volume of data associated with pregnancy-related studies, adds an additional overhead to classical statistical methods, to meander this inherent problem various computational workflows have been developed using well known computational architecture. Tasks like association and relationship of variables(metabolites/microbiome/proteome) to the outcome can be achieved with a higher degree of accuracy using the well recognized computational workflows. In the current times, several researchers are also utilizing neural networks for prediction and association testing. The interconnection between various features are masked to several classical statistical methods, but using deep neural networks, we unearth this complex relationship and accordingly adjust the weights, which inturn bolsters the prediction outcomes. The added advantage of using the computational processes is that it renders parallelization in the workflow which drastically reduces the time required and creates automated steps, so no or nominal user input is required to reach the end goal.

Statistical methods used in the reference articles of this review are presented in Table 1. As omics technologies evolve, fresh and novel statistical and machine-learning techniques are needed to analyze data while considering the property of and structure of omics data. The most frequent statistical method is the T-test. Receiver Operating Characteristic (ROC) Analysis is frequently mentioned in the articles to evaluate the performance of the tests and the accuracy of the statistical models. The most popular kind of regression is the logistic regression model.

**Table 1:** Statistical methods and machine learning techniques are used in pregnancy literature

Statistical methods	Studies
T-test	Preeclampsia [15, 17, 22, 24, 30] | Metabolic syndrome [46] | Gestational diabetes[27, 48] Placental abruption [20] | Fetal growth restriction [23] | Asthma in offspring [56] Alternations of breastmilk components of COVID-19 [51]
Fisher's Exact Test	Preeclampsia [21, 22, 24, 30] | Fetal growth restriction [25] | Gestational diabetes [26] Preterm delivery [29] | Alternations of breastmilk components of COVID-19 [51] Asthma in offspring [54, 56]
Mann Whitney Rank Sum Test	Preeclampsia [17, 21, 28, 30] | Obesity and overweight [45] | Fetal growth restriction [25] Gestational diabetes [26] | Preterm delivery [29]
Chi-squared test (include Wald test)	Preeclampsia [13, 24, 30] | Placental abruption [20] | Preterm delivery [29] Asthma in offspring [53, 54]
ANOVA	Preeclampsia [13, 22, 28, 30] | Placental abruption [18] | Metabolic syndrome [46] Asthma in offspring [53]
ROC analysis	Placental abruption [20] | Preeclampsia [21, 22, 28] | Gestational diabetes [26, 27] Preterm delivery [29]
Logistic regression model (including Hosmer–Lemeshow Goodness-of-Fit Test)	Placental abruption [18, 20] | Gestational diabetes [26] | Asthma in offspring [53, 56]
Spearman rank correlations	Preeclampsia [24, 28] | Metabolic syndrome [46]
Post hoc analyses	Respiratory syncytial virus [4] | Preeclampsia [22, 30] | Asthma in offspring [53]
Linear regression model (including mixed effect model)	Maternal cardiovascular adaptation [3] | Preeclampsia [24] | Asthma in offspring [54]
Kaplan–Meier curve	Asthma in offspring [53, 54]
Kolmogorov–Smirnov test	Preeclampsia [22] | Gestational diabetes [27]
Principal component analysis (PCA)	Preeclampsia [13] | Metabolic dynamics of timing of delivery [19]
Cox regression	Asthma in offspring [53, 54]
Pearson correlation coefficient	Obesity and overweight [45]

### Changing physiology in pregnancy: challenges to study

Implementing multi-omics techniques in the study of pregnancy requires an understanding of the scientific, logistical, and practical challenges of studying pregnancy. Bias is inherent in the study of pregnancy, beginning with the non-random, self-selecting sample of individuals who become pregnant and choose to enroll in prospective studies. With approximately half of US and world pregnancies being unplanned [57], many individuals may be unaware that they are pregnant until well into the first trimester, following the occurrence of significant fetal development and/or spontaneous fetal demise. Those aiming to apply omics approaches to understand physiology further or diagnose pregnancy-related diseases can appreciate the challenges posed by these unique aspects of the pregnancy phenotype.

**Fig. 3:**
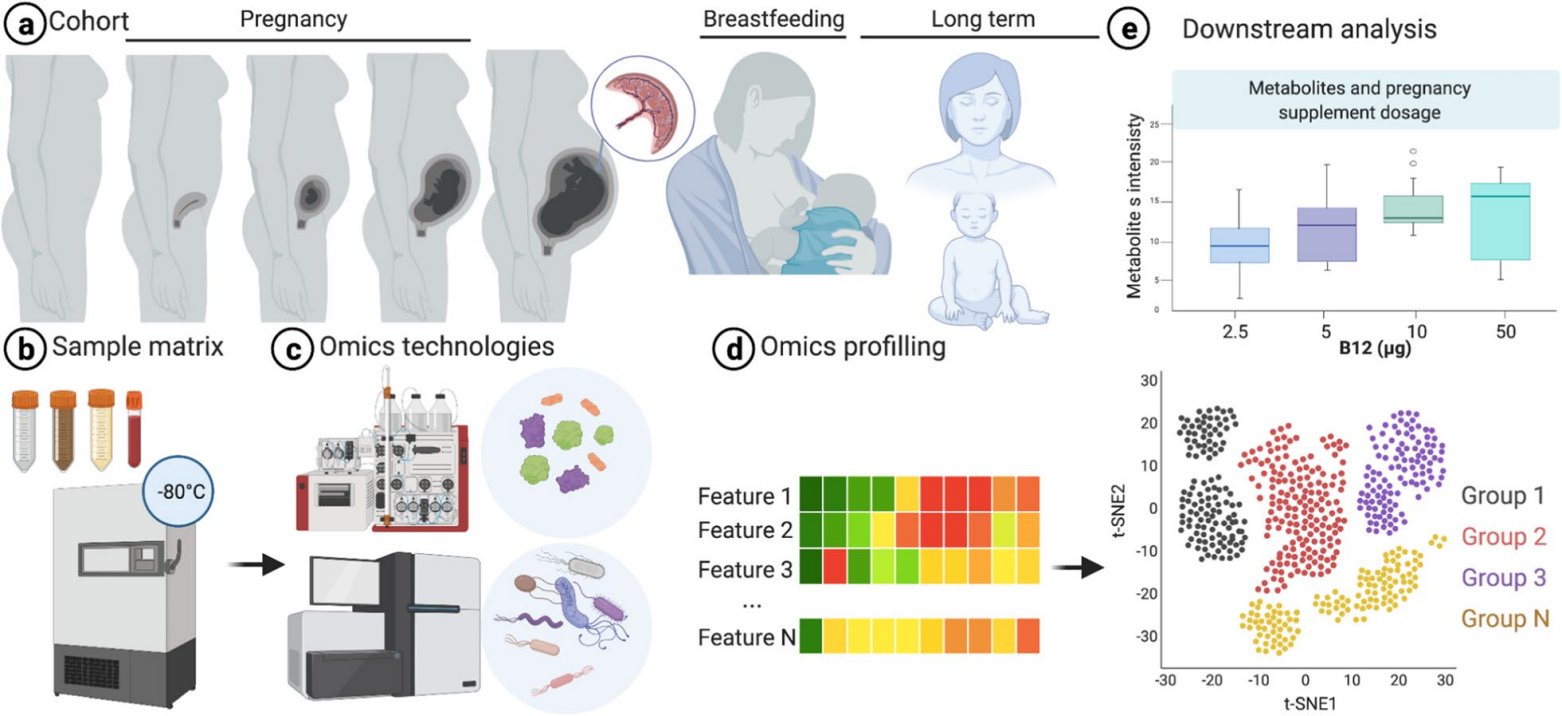
Overview workflow of multi-omics in pregnancy. **a**, study design is a key feature in a project to collect samples and measure omics to investigate birth development, pregnancy physiology and pathophysiology, and long-term health effect at molecular levels. **b**, various sample matrices can be collected from individuals (mothers and babies), including breast milk, stool, urine, and blood. **c**, **d**, omics technologies such as Next Generation Sequencing (NGS) for genome and metagenome data, and LC–MS for metabolomics and proteomics profiling enable measuring millions of biomarkers of health. **e**, downstream analysis includes processing data and applying computational approaches to discover patterns that explain molecular dynamics of pregnancy biology, causality, and correlations. This figure is created with BioRender.com

Ideal prospective cohorts of pregnant people generally require longitudinal profiling of women of reproductive age prior to pregnancy and throughout the pregnancy course, including the periparturient and postpartum period (Fig. 3a). Comprehensive sampling of various matrices [e.g., whole blood and its constituent cell types and circulating extracellular vesicles, 24-h urine, fecal sampling, and tissue biopsies (Fig. 3b)] enables investigation of various health conditions and related complexity. Idealized cohorts could follow individuals through multiple pregnancies, documenting parity and inter-birth intervals, and characterize the broader exposome through self-report and medical chart review (e.g., diet, mental health, infectious diseases). Such designs would allow for multi-omic analyses applied to a variety of tissue matrices to isolate (Fig. 3c, d), in combination with preclinical model systems, pregnancy-specific alterations (Fig. 3e). In addition, data curation and downstream analysis need to consider specific properties of omics data such as high-dimensionality, zero-inflated, and collinearity to increase the biology-to-noise ratio in data analysis.

While there are studies for some human diseases, such as inflammatory bowel disease [39], that aim to encapsulate all the omics technology in a single cohort. Thus, the application of individual or multi-omics approaches during pregnancy has been limited to providing select inferences. Nevertheless, successes of omics are evident in the literature.

## Conclusions

This review summarizes much of the recent work that has been done on pregnancy using omics tools. Omics technologies and techniques are useful tools in molecular investigation of pregnancy epidemiology; the tools are constantly evolving and promising for future research. Single-cell genomics, shotgun sequencing metagenomics, LC–MS metabolomics, and proteomics are rapidly increasing in scientific literature. However, there are few pregnancy studies that have utilized omics technologies to investigate pregnancy physiology through the lifecycle, to probe the effect of infection, poor nutrition, or environmental exposures during pregnancy, or as a complement to ongoing randomized trials of preventions and therapeutics. While there has been great progress with omics technologies, challenges remain.

Pregnancy is highly dynamic and lacks a clear, steady state. For example, plasma volume expands throughout pregnancy [58], which may affect omics interpretation in longitudinal studies. There is apparently no significant longitudinal study of pregnancy omics across maternal, placental, and fetal compartments. Designing studies with the appropriate comparison groups and optimal exposure assessment are also challenging in pregnancy. For example, it can be challenging to obtain exposure information pre-pregnancy and throughout pregnancy. Proper comparison groups might include non-pregnant female peers or might include pregnant people without exposure of interest. Important confounding factors such as fetal sex and genotype, parity, and interbirth intervals that likely influence metabolism need to be considered in pregnancy studies.

In future studies, we suggest including multi-omics to generate a comprehensive snapshot from various biological angles (e.g., transcriptome, metabolomics). Study design is a critical consideration for incorporating omics components, and if there are limits in collecting samples, then elements such as number of samples and time of collection should be carefully chosen. Omics can be used for risk prediction modeling and prognostic studies. For example, metabolic of placenta can give a better understanding of health outcomes and assist disease diagnostics during pregnancy. Pregnant women's characteristics (e.g., age, race, and country) need to be considered in future studies. Omics are useful tools for investigating fundamental biology of pregnancy and enable researchers to consider interaction between millions of omics features to characterize biology of pregnancy and improve healthy pregnancy.

### Supplementary Information

**Additional file 1: Table S1.** For investigating literature on pregnancy omics, we added “AND Pregnancy” to our regular expression search. **Figure S1.** Current important scientific directions of omics utilization in pregnancy research. Abstract of 219 papers with expression search of omics and pregnancy, and condition we process and from 1385 extracted Scientific keywords from abstracts that occurred with pregnancy and omics we show 97 keywords with least 5 co-occurrence. The co-occurrence link with other keywords also is measured and shown as links between keywords. Colors represent year of publication. The network analysis was performed by VOSviewer[2]. Nodes are keywords that are linked by edges for their co-occurrence. Edges reflect the number of co–co-occurrence of keywords in publications used in the analysis. Each color refers to a cluster of keywords that co-occurred in publications. **Figure S2**. Current important scientific directions of omics utilization in all research domain literature. Abstract of 18,502 papers with expression search of omics, and condition we process and from 1385 extracted Scientific keywords from abstracts that occurred with omics we show 214 keywords with least 100 co-occurrence. The co-occurrence link with other keywords also is measured and shown as links between keywords. Colors represent years of publications. The network analysis was performed by VOSviewer[2]. Nodes are keywords that are linked by edges for their co-occurrence. Edges reflect the number of co–co-occurrence of keywords in publications used in the analysis. Each color refers to a cluster of keywords that co-occurred in publications.

**Additional file 2.** Comprehensive literature review of omics research in pregnancy.

## Data Availability

Not applicable.

## Abbreviations

GC–MS: Gas chromatography-mass spectrometry
LC–MS: Liquid chromatography-mass spectrometry
RNA: Ribonucleic acid
PE: Preeclampsia
sFLT1: fms-like tyrosine kinase 1
sENG: Soluble endoglin
VEGF: Vascular endothelial growth factor
PLGF: Placental growth factor
PA: Placental abruption
SERPINA3: α-1-antichymotrypsin
FGR: Fetal growth restriction
PAPP-A: Pregnancy-associated plasma protein A
MOMS-PI: Multi-omic microbiome study—pregnancy initiative
BVAB: Bacterial vaginosis-associated bacteria
NADPH: Nicotinamide adenine dinucleotide phosphate
LDL: Low-density lipoprotein
GDM: Gestational diabetes mellitus
n-3 LCPUFAs: n-3 long-chain polyunsaturated fatty acids
PUFA: Polyunsaturated fatty acid
PC: Phosphatidylcholine
UPLC-MS/MS: Ultra-performance liquid chromatography-tandem mass spectroscopy
ROC: Receiver operating characteristic
PCA: Principal component analysis
NGS: Next generation sequencing
