# Supplementary material for: Molecular epidemiology of pregnancy using omics data: advances, success stories, and challenges
Source: J Transl Med. 2024 Jan 26;22:106. doi: 10.1186/s12967-024-04876-7 (PMC10821542; doi:10.1186/s12967-024-04876-7)
Supplement: Supplementary file 1 — Additional file 1: Table S1. For investigating literature on pregnancy omics, we added “AND Pregnancy” to our regular expression search. Figure S1. Current important scientific directions of omics utilization in pregnancy research. Abstract of 219 papers with expression search of omics and pregnancy, and condition we process and from 1385 extracted Scientific keywords from abstracts that occurred with pregnancy and omics we show 97 keywords with least 5 co-occurrence. The co-occurrence link with other keywords also is measured and shown as links between keywords. Colors represent year of publication. The network analysis was performed by VOSviewer[2]. Nodes are keywords that are linked by edges for their co-occurrence. Edges reflect the number of co–co-occurrence of keywords in publications used in the analysis. Each color refers to a cluster of keywords that co-occurred in publications. Figure S2. Current important scientific directions of omics utilization in all research domain literature. Abstract of 18,502 papers with expression search of omics, and condition we process and from 1385 extracted Scientific keywords from abstracts that occurred with omics we show 214 keywords with least 100 co-occurrence. The co-occurrence link with other keywords also is measured and shown as links between keywords. Colors represent years of publications. The network analysis was performed by VOSviewer[2]. Nodes are keywords that are linked by edges for their co-occurrence. Edges reflect the number of co–co-occurrence of keywords in publications used in the analysis. Each color refers to a cluster of keywords that co-occurred in publications. [file 12967_2024_4876_MOESM1_ESM.pdf]

Supplement

Molecular epidemiology of pregnancy using omics data: advances, success stories, and challenges

Authors: Ali Rahnavard[1,2,*], Ranojoy Chatterjee[1,2], Hui Wen[1,2], Clark Gaylord[1,2], Sabina Mugusi[3], Kevin C Klatt[4], Emily R. Smith[5,6,*]

Affiliations:

[1]Computational Biology Institute, Milken Institute School of Public Health, The George Washington University, Washington, DC 20052 USA.

[2]Department of Biostatistics and Bioinformatics, Milken Institute School of Public Health, The George Washington University, Washington, DC 20052 USA.

[3]Department of Clinical Pharmacology, Muhimbili University of Health and Allied Sciences, Dar es Salaam, Tanzania.

[4]Nutritional Sciences & Toxicology, University of California, Berkeley, CA 94720, USA.

[5]Department of Global Health, The Milken Institute School of Public Health, The George Washington University, Washington, DC 20052 USA.

[6]Department of Exercise and Nutrition Sciences, The Milken Institute School of Public Health, The George Washington University, Washington, DC 20052 USA.

*Correspondence to rahnavard@gwu.edu and emilysmith@gwu.edu

Omics literature processing: We used the PUBMED database[1] on February 25th, 2022, and to find original researches, we filtered for "*Journal Article"* and used Clinical Trial (Phase I, II, III, and IV) Controlled Clinical Trial, Journal Article, Multicenter Study, Observational Study, and Randomized Controlled Trial. We also filtered species and language by only selecting "human" and "english". The search terms that were associated with each category are given below in **STable 1**. For investigating literature on pregnancy omics, we added "AND Pregnancy" to our regular expression search.

Omics	Studies		
Metabolomics	(metabolom*) AND (NMR)	(metabolom*) AND (GC-MS)	(metabolom*) AND (LC-MS)
Metagenomics	(metagenom*) AND (16S)	(metagenom*) AND (18S)	(metagenom*) AND (Shotgun)
Genomics	(genom*) AND (Microarray)	(genom*) AND (NGS)	(genom*) AND (qPCR)
Epigenomics	(epigenom*) AND (ATAC-SEQ)	(epigenom*)AND (CHIp-SEQ)	(epigenom*) AND (HI-C)
Proteomics	(proteom*) AND (LC-MS)	(proteom*) AND (NMR)	(proteom*) AND (GC-MS)
Viromics	(virom*) AND (NGS)	(virom*) AND (HTS)	

STable 1: Search string used to capture publications for each omic.

Network analysis and data mining of literature:

Expression search: 1) (OMICS) AND (PREGNANCY) resulted in 219 papers (**SFig. 1**) colored by year of publication and 2) OMICS search results for 18,502 papers. Omics literature had 39,489 keywords 214 of them co-occurred with the omics keyword at least 100 times. Our network analysis using VOSviewer[2] identified 6 clusters (each specified with different colors (**SFig. 2**). Papers filtered for categories including Clinical Trial, Clinical Trial, Phase I, Clinical Trial, Phase II, Clinical Trial, Phase III, Clinical Trial, Phase IV, and only Journal Article, Randomized Controlled Trial, and language English, which resulted in 18,502 papers.

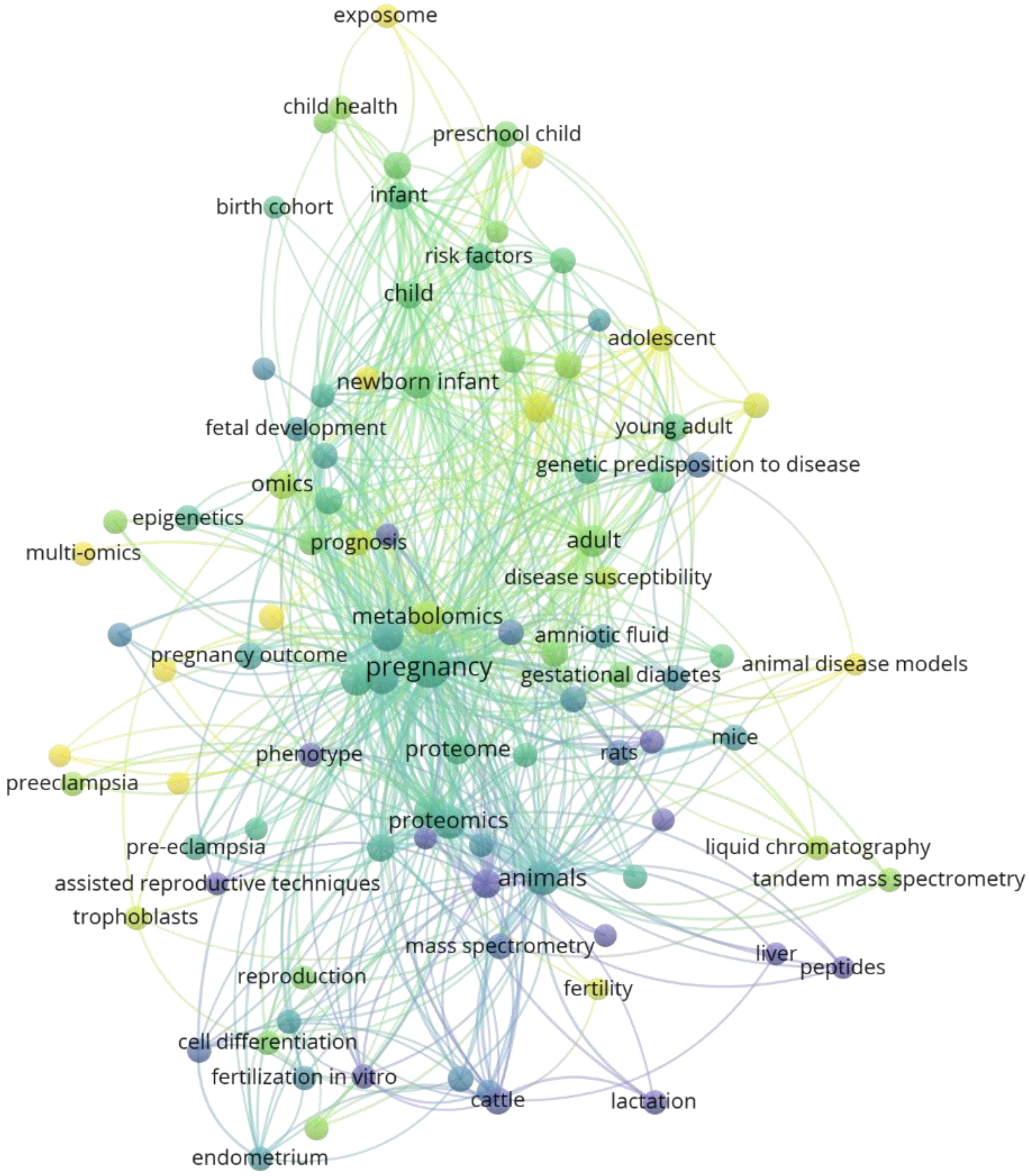

SFigure 1: Current important scientific directions of omics utilization in pregnancy research. Abstract of 219 papers with expression search of omics and pregnancy, and condition we process and from 1385 extracted Scientific keywords from abstracts that occurred with pregnancy and omics we show 97 keywords with least 5 co-occurrence. The co-occurrence link with other keywords also is measured and shown as links between keywords. Colors represent year of publication. The network analysis was performed by VOSviewer[2]. Nodes are keywords that are linked by edges for their co-occurrence. Edges reflect the number of co-co-occurrence of keywords in publications used in the analysis. Each color refers to a cluster of keywords that co-occurred in publications.

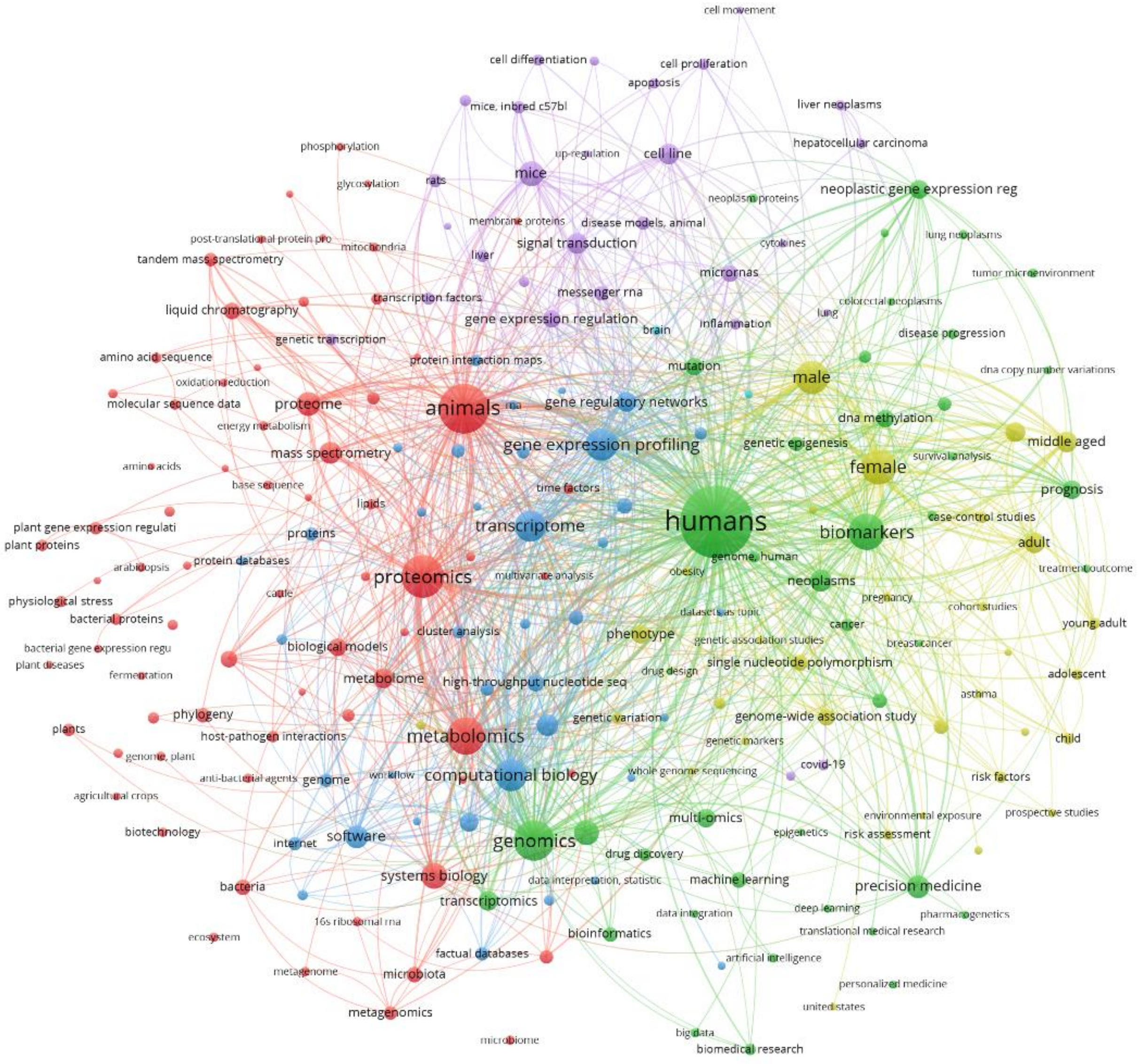

SFigure 2: Current important scientific directions of omics utilization in all research domain literature. Abstract of 18,502 papers with expression search of omics, and condition we process and from 1385 extracted Scientific keywords from abstracts that occurred with omics we show 214 keywords with least 100 co-occurrence. The co-occurrence link with other keywords also is measured and shown as links between keywords. Colors represent years of publications. The network analysis was performed by VOSviewer[2]. Nodes are keywords that are linked by edges for their co-occurrence. Edges reflect the number of co-co-occurrence of keywords in publications used in the analysis. Each color refers to a cluster of keywords that co-occurred in publications.

Supplementary references

1. PubMed [Internet] [Internet]. Bethesda (MD): National Library of Medicine (US), National Center for Biotechnology Information; 2004 [cited 2022 Mar 10]. Available from: https://www.ncbi.nlm.nih.gov/pubmed/

2. Perianes-Rodriguez A, Waltman L, van Eck NJ. Constructing bibliometric networks: A comparison between full and fractional counting. J Informetr. 2016;10:1178–95.
